# Astrocyte Control of Zika Infection Is Independent of Interferon Type I and Type III Expression

**DOI:** 10.3390/biology11010143

**Published:** 2022-01-15

**Authors:** Mithun Das, Monique L. Smith, Tomomi Furihata, Subir Sarker, Ross O’Shea, Karla J. Helbig

**Affiliations:** 1 Department of Physiology, Anatomy and Microbiology, La Trobe University, Melbourne, VIC 3086, Australia; mithundaskubge06@gmail.com (M.D.); m.smith3@latrobe.edu.au (M.L.S.); S.sarker@latrobe.edu.au (S.S.); r.oshea@latrobe.edu.au (R.O.); 2 Laboratory of Clinical Pharmacy & Experimental Therapeutics, School of Pharmacy, Tokyo University of Pharmacy and Life Sciences, Tokyo 192-0392, Japan; tomomif@toyaku.ac.jp

**Keywords:** Zika, astrocyte, interferon, antiviral, host response

## Abstract

**Simple Summary:**

Zika virus (ZIKV) is a mosquito-borne virus first isolated from the Zika forest, Uganda, in 1947, which has been spreading across continents since then. We now know ZIKV causes both microencephaly in newborns and neurological complications in adults; however, no effective treatment options have yet been found. A more complete understanding of Zika-infection-mediated pathogenesis and host responses is required to enable the development of novel treatment strategies. In this study, efforts were made to elucidate the host responses following Zika virus infection using several astrocyte cell models, as astrocytes are a major cell type within the central nervous system (CNS) with significant antiviral ability. Our data suggest that astrocytes can resist ZIKV both in an interferon type I- and III-independent manner and suggest that an early and more diverse antiviral response may be more effective in controlling Zika infection. This study also identifies astrocyte cellular models that appear to display differential abilities in the control of viral infection, which may assist in the study of alternate neurotropic virus infections. Overall, this work adds to the growing body of knowledge surrounding ZIKV-mediated cellular host interactions and will contribute to a better understanding of ZIKV-mediated pathogenesis.

**Abstract:**

Zika virus (ZIKV) is a pathogenic neurotropic virus that infects the central nervous system (CNS) and results in various neurological complications. Astrocytes are the dominant CNS cell producer of the antiviral cytokine IFN-β, however little is known about the factors involved in their ability to mediate viral infection control. Recent studies have displayed differential responses in astrocytes to ZIKV infection, and this study sought to elucidate astrocyte cell-specific responses to ZIKV using a variety of cell models infected with either the African (MR766) or Asian (PRVABC59) ZIKV strains. Expression levels of pro-inflammatory (TNF-α and IL-1β) and inflammatory (IL-8) cytokines following viral infection were low and mostly comparable within the ZIKV-resistant and ZIKV-susceptible astrocyte models, with better control of proinflammatory cytokines displayed in resistant astrocyte cells, synchronising with the viral infection level at specific timepoints. Astrocyte cell lines displaying ZIKV-resistance also demonstrated early upregulation of multiple antiviral genes compared with susceptible astrocytes. Interestingly, pre-stimulation of ZIKV-susceptible astrocytes with either poly(I:C) or poly(dA:dT) showed efficient protection against ZIKV compared with pre-stimulation with either recombinant IFN-β or IFN-λ, perhaps indicating that a more diverse antiviral gene expression is necessary for astrocyte control of ZIKV, and this is driven in part through interferon-independent mechanisms.

## 1. Introduction

Zika virus (ZIKV), a recent global health concern, causes neurological complications including congenital brain abnormalities [1], microcephaly in infants [2,3], Guillain–Barré syndrome (GBS) [4], meningoencephalitis and myelitis [5]. ZIKV is a member of the *Flaviviridae* family and the genus *Flavivirus*, and belongs to the larger group of arboviruses (arthropod-borne viruses), with the Aedes genus of mosquitoes being the primary route of ZIKV transmission [6,7,8]. Other than arthropods, ZIKV can also be transmitted through other routes including mother to child, and sexual and blood transfusion [9,10]. Regardless of transmission strategy, ZIKV commonly infects the central nervous system (CNS), which has been found to be linked with various neurological complications, including infection of foetal and adult brain causing microcephaly and GBS [3,11,12,13].

The CNS is made of numerous cell types, with astrocytes being abundant, heterogeneous neuroglial cells that provide crucial support for the functioning of neurons and contribute to brain function, including homeostasis and defense of the CNS [14,15]. As astrocytes are known to be one of the dominant producers of antiviral cytokines, including type I IFN, they are likely to be key players in an effective antiviral response to ZIKV and other viruses [16,17,18]. Multiple studies have demonstrated that some astrocytes can be infected by neurotropic flaviviruses, including tick-borne encephalitis virus (TBEV), Zika virus (ZIKV), West Nile virus (WNV), and Japanese encephalitis virus (JEV) [19]. However, many studies have also shown astrocytes to be highly resistant to these viruses [16,17,18], with differences in the morphology, gene expression, and function within astrocyte cell subsets suggested as possible reasons for this discrepancy [16,17,18,19,20]. As an example, cerebellar astrocytes have been found to be key responders to WNV infection while cerebral cortical astrocytes are not as efficient, highlighting the existence of distinct innate immune responses in astrocytes from evolutionarily disparate regions of the CNS [21]. Additionally, a recent study added another dimension to the functional feature of antiviral immunity in the CNS by demonstrating that a small sub-set of interferon-stimulated genes restricted the replication of neurotropic flaviviruses in the CNS in a region- and cell-type-specific manner [22].

There are differing opinions in the current scientific literature regarding the ability of astrocytes to control ZIKV infection [23,24,25,26]. Human astrocyte cell models are scarce, and in this study, we utilised a previously existing human astrocytic (astrocytoma) cell line (CCF-STTG1) [27,28,29] and two newly developed human immortalised primary astrocyte cell lines, foetal hTERT (abmGood, Richmond, BC, Canada) and foetal HASTRci35 [30,31]. Although these cell models were previously used in brain physiology and cancer-related research [31,32,33,34,35,36], their use in antiviral immunity is limited. This work sought to analyse the antiviral response of multiple available human astrocyte cell models following Zika virus infection to understand the variability within astrocyte cell subsets in control ZIKV infection, as well as determine the ability of the pre-stimulation of the innate immune response to impact ZIKV infection in these model systems.

## 2. Materials and Methods

### 2.1. Tissue Culture

The CCF-STTG1 astrocyte cell line (The European Collection of Authenticated Cell Cultures (ECACC)) and the newly developed primary immortalised human astrocyte HASTR/ci35 cell line were cultured in RPMI Medium 1640 (1X) (Gibco, Waltham, MA, USA) containing 10% FBS,100 I.U./mL penicillin and 100 μg/mL streptomycin (Sigma-Aldrich, St. Louis, MO, USA) and 2.5 ug/mL Plasmocin (InvivoGen, San Diego, CA, USA). Vero cells and primary immortalised human astrocytes, foetal-hTERT (abmGood, Richmond, BC, Canada) were cultured in Dulbecco’s Modified Eagle Medium (Gibco, Waltham, MA, USA) containing 10% foetal bovine serum (FBS), 100 I.U./mL penicillin and 100 μg/mL streptomycin and 2.5 µg/mL Plasmocin. C6/36 cells were cultured using Minimum Essential Medium Eagle (Sigma-Aldrich, St. Louis, MO, USA) containing 10% FBS and 100 I.U./mL penicillin and 100 μg/mL streptomycin. The Mouse hybridoma B lymphocyte cells were purchased from ATCC and used to produce flavivirus group antigen Antibody (D1-4G2-4-15 (4G2)). They were cultured using Hybri-Care Medium, containing 10% FBS, 1.5 g/L sodium bicarbonate and 100 I.U./mL penicillin and 100 μg/mL streptomycin. All the cells were cultured by incubating at 37 °C and 5% CO_2_ conditions, except the C6/36 cell line, which was incubated at 28 °C with no CO_2_.

### 2.2. Pre-Stimulation of Cells

For in vitro studies, cells were seeded at a concentration of 0.5 × 10^5^, 1 × 10^5^ and 2 × 10^5^ per well of 24-, 12- and 6-well plates, respectively, unless indicated otherwise, and incubated for 24 h at 37 °C in 5% CO_2_ before adding stimulants. To elucidate the ability of interferons (IFNs) and viral mimics to control ZIKV, CCF-STTG1 and HASTR/ci35 cells were pre-treated with human IFN type I (IFN-β) (PBL Assay Science, Piscataway, NJ, USA), type III IFN (IFN-λ1/IL-29) (R&D System, Minneapolis, MN, USA), dsRNA (poly(I:C)) (Invivogen, San Diego, CA, USA) and dsDNA (poly(dA:dT)) (Sigma-Aldrich, St. Louis, MO, USA). IFN-β and IFN-λ1/IL-29 were topically added to the cells while poly(I:C) and poly(dA:dT) were transfected into the cells using DMRIE-C Transfection Reagent (Thermo Fisher Scientific, Inc., Waltham, MA, USA) following the manufacturer’s protocol. Stimulated or transfected cells were incubated for 18 h, followed by ZIKV infection at an MOI of 0.1.

### 2.3. Zika Virus Propagation

Zika virus African strain MR766 (Uganda, 1947) and Asian strain PRVABC59 (Puerto Rico, 2015) were propagated in C6/36 cells by infecting cells at a multiplicity of infection (MOI) of 0.1. Cells were seeded in tissue culture flasks at a concentration of 25,000 cells/cm^2^ and incubated for 24 h. Cells with a ~70% confluency were infected with ZIKV stock at an MOI of 0.1 and incubated for 4 days at 28–29 °C temperature in CO_2_-free condition. At day 4 post-infection (p.i.), or when a cytopathic effect (CPE) appeared, the supernatant was harvested and filtered using a 0.45 μm syringe. Filtered supernatant containing viruses was stored in −80 °C for infection experiments.

### 2.4. Zika Virus Titration by Plaque and Focus Forming Unit (FFU) Assays

Zika virus infectivity was determined by plaque assay using Vero cells. Briefly, Vero cells were seeded in 6-well plates at a concentration of 1 × 10^6^ cells/well to attain 70% confluency after 24 h of incubation. Virus stocks were serially diluted from 10^−1^ to 10^−9^ (10-fold serial dilutions of the virus) in serum-free DMEM, then cells were infected with 800 μL of serially diluted virus containing supernatants for 1 h at 37 °C. Supernatants were then replaced with a 2 mL overlay of complete media containing 1.5% (*w*/*v*) carboxymethyl cellulose (CMC) (Sigma-Aldrich, St. Louis, MO, USA). Cells were incubated for 5–7 days at 37 °C temperature in 5% atmospheric CO_2_ conditions until plaques appeared. Cell monolayers were then fixed by adding 1–2 mL of 10% formalin and incubating for 1 h. The CMC overlay was then gently removed, and cells were stained with 900 μL 1–2% crystal violet (diluted in 10% ethanol) to visualise plaques. Crystal violet stain was removed by washing the well multiple times with ddH_2_O. Focus-forming unit assays were performed in duplicate by seeding 2 × 10^4^ HeLa cells/well in a 96-well plate 24 h prior to the addition of serial diluted viral containing supernatant. Viral containing supernatant was washed off the plates with PBS twice, prior to the replacement of complete medium. Three days post-infection, cells were washed with PBS, fixed and stained as stated below. FFU was calculated by counting visible fluorescent foci of at least 4 cells in the serial dilution containing between 10 and 30 ffu.

### 2.5. Microscopy and Antibodies

For in vitro studies, cells were seeded at a concentration of 0.5 × 10^5^, 1 × 10^5^ and 2 × 10^5^ per well of 24-, 12- and 6-well plates, respectively, unless indicated. For the ZIKV infection kinetics study using fluorescence microscopic studies, cells were seeded at the same time as above, and fixed with acetone:methanol or 4% paraformaldehyde for staining at the specified time of post-infection. Cells were either lysed to analyse mRNA expression and viral RNA replication or fixed with acetone:methanol (1:1) to stain for ZIKV antigen using anti-4G2 hybridoma fluid against flavivirus group antigen. Cells were then incubated with goat anti-mouse IgG (H + L) cross-adsorbed secondary antibody, Alexa Fluor 488 (Thermo Fisher Scientific, Waltham, CA, USA) and nuclei stained using DAPI diluted in ddH_2_O at a ratio of 1:10,000 (Sigma-Aldrich, St. Louis, MO, USA). Cells were visualised on a Nikon ECLIPSE Ti inverted fluorescence microscope and images were captured using NIS Elements software (Nikon, Minato City, Tokyo, Japan). Images were processed using NIS Elements software and biological image-processing software ImageJ (National Institutes of Health, Bethesda, MD, USA) [37].

### 2.6. RNA Extraction, cDNA Preparation, RT-qPCR and ELISA

For quantitative mRNA expression studies, total cellular RNA was extracted using TRIsure Reagent (Bioline, London, England, UK) as per the manufacturer’s instructions, the RNA pellet was dissolved in 20–30 μL of DEPC-treated RNase-free dH_2_O and stored at −80 °C until use. First-strand cDNA synthesis was performed using a Tetro cDNA Synthesis Kit (Bioline, London, England, UK) as per manufacturer’s instructions. cDNA samples were diluted to a final volume of 80 μL with DEPC-treated H_2_O and stored in a −20 °C freezer for long-term storage. Quantitative real-time PCR (RT-qPCR) was performed to quantitate the relative levels of mRNA of the targeted genes in comparison with the housekeeping gene 36b by comparing relative Cq values. RT-qPCR reactions were performed using SensiFAST SYBR^®^ No-ROX Kit (Bioline, London, England, UK). The reactions consisted of 5 μL of the diluted cDNA from the previous step, 5 μL of SensiFAST SYBR No-ROX master mix and 6 pmol of each forward and reverse primer. DEPC-treated nuclease-free H_2_O was added to achieve a 20 μL final volume of the reaction mix. All samples were analysed in duplicate. A CFX Connect Real-Time Detection System (BioRad, Hercules, CA, USA) coupled with the CFX manager software was used to control the PCR reaction, and the PCR program comprised denaturation at 95 °C for 2 min followed by 39 cycles of 95 °C for 5 s and 60 °C for 20 s. A step of 65 °C for 5 s followed by a final step of 95 °C for 50 s was performed to facilitate the melting curve. Primer sequences or oligos used for RT-qPCR were purchased from Geneworks, Thebarton, South Australia, Australia or Integrated DNA Technologies, Inc. (IDT), Coralville, IA, USA and are listed in Table S1. The IFN-β ELISA’s were performed as per manufacturer’s instructions using a 1:2 dilution of culture supernatant at the indicated times (R&D systems, Minneapolis, MN, USA).

### 2.7. Statistical Analysis

Infection experiments were performed independently and in triplicate, and all experimental mRNA expression analysis was performed in at least triplicate. Experimental quantitative data were statistically analysed and graphed using GraphPad Prism 8 (Graphpad Software Inc., San Diego, CA, USA), and are presented with means and the standard error of the mean (SEM). Statistical analyses within different biological conditions were conducted using parametric one-way or two-way ANOVA comparisons with Tukey or Sidak’s post hoc tests. Tukey’s post hoc test was considered when performing all possible pairwise comparisons amongst a group of means and Sidak’s post hoc test was considered when performing planned orthogonal comparisons. T-test was performed to analyse significant difference only between two experimental conditions. The level of statistical confidence was set at *p* < 0.05.

## 3. Results

### 3.1. Variability in ZIKV Load within Different Astrocyte Cell Models

**Figure 1:**
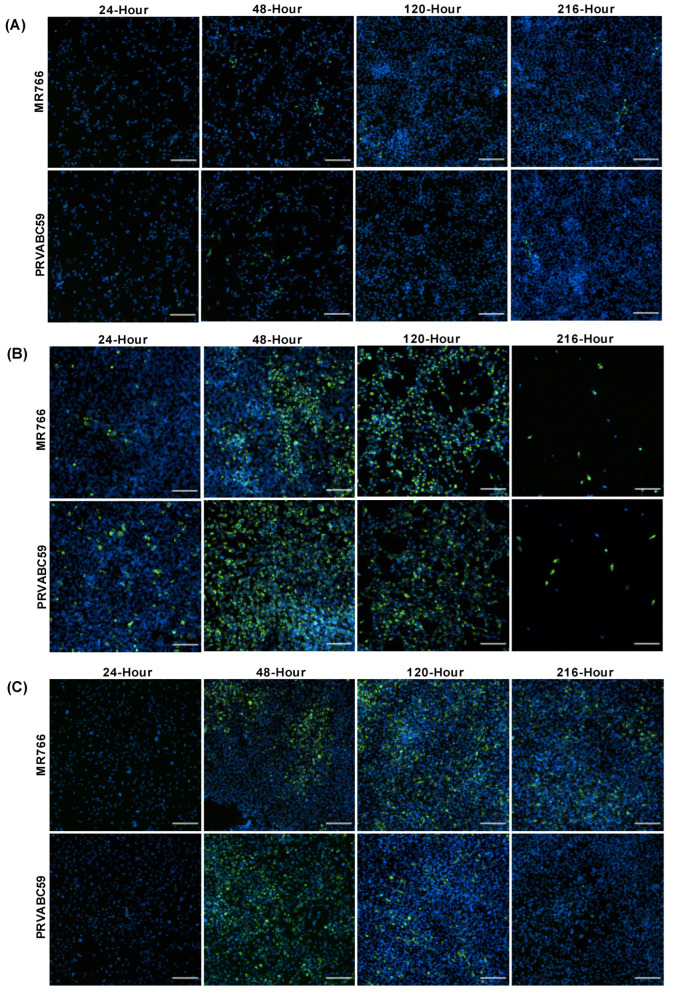
Zika virus load in different astrocyte cell lines. Cells were infected with ZIKV strains MR766 and PRVABC59 at a multiplicity of infection (MOI) of 0.1. (**A**–**C**) Staining of Zika virus envelope (Env) protein in CCF-STTG1 (**A**), HASTR/ci35 (**B**) and foetal-hTERT (**C**) astrocyte cell lines. HASTR/ci35 cells showed higher viral load followed by complete cell death. The 2 rows within each set of 8 panels correspond to merged images stained with 4G2 antibody to detect ZIKV envelope protein Env (stained green) and DAPI stain for cell nuclei (stained blue). Scale bars represent 200 µm.

To determine ZIKV infection efficiency in different astrocyte cell models, CCF-STTG1, hTERT and HASTR/ci35 astrocyte cells were infected with both the African (MR766) and Asian (PRVABC59) ZIKV strains at an MOI of 0.1. CCF-STTG1 appeared to have very low levels of ZIKV infection throughout the time kinetics experiments with only small pockets of cells infected at each timepoint and no apparent cell death (Figure 1A). In contrast, hTERT and HASTR/ci35 cells demonstrated significant levels of ZIKV Env protein at 48 h post-infection (Figure 1B,C). HASTR/ci35 cells displayed significant cell death at the 120 h timepoint p.i., with almost complete cell death observed at 216 h (Figure 1B); however, hTERT cells did not show considerable cell death, and displayed comparatively fewer ZIKV-positive (green) cells at the later timepoints, especially following infection with the PRVABS59 ZIKV strain (Figure 1C).

**Figure 2:**
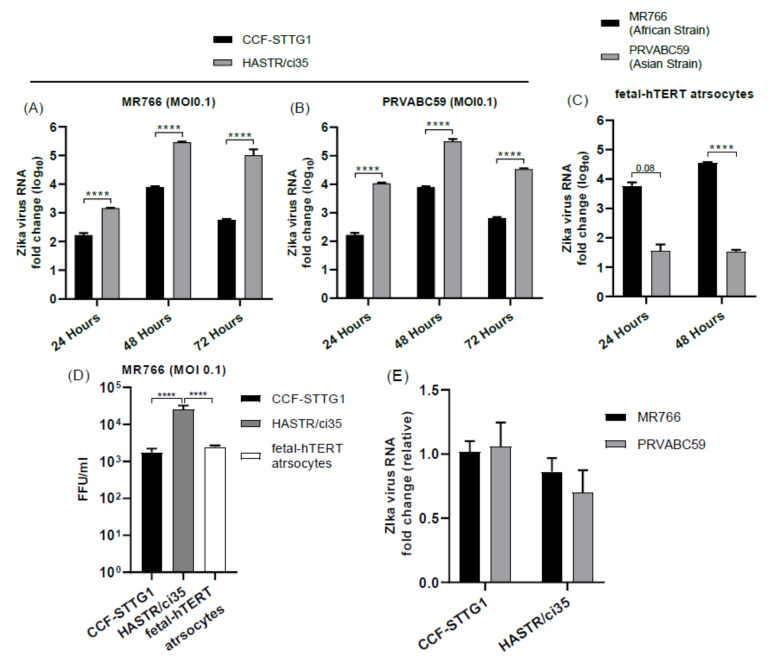
Zika virus load in different astrocyte cell lines. Cells were infected with ZIKV strains MR766 and PRVABC59 at a multiplicity of infection (MOI) of 0.1. (**A**–**C**) Quantification of Zika virus RNA load in astrocyte cell subtypes using relative quantitative PCR. CCF-STTG1 astrocyte cells presented lowest ZIKV load at all timepoints compared with HASTR/ci35 and foetal-hTERT astrocyte cells. HASTR/ci35 cells showed higher viral RNA load (**A**–**C**) despite similar level viral entry compared with CCF-STTG1 (**E**). Focus-forming unit (FFU) assay was performed on supernatants collected at 24 h post-infection to assess the infection ability of the virus particles shed by the astrocyte cell lines. HASTR/ci35 astrocytes shed high-level active MR766 virus particles at 24 h compared with CCF-STTG1 and foetal-hTERT astrocytes (**D**). Data were normalised to the 36b housekeeping gene and expressed as a fold change relative to ZIKV RNA load at 6 h timepoints relative to mock-infected control (**A**–**C**) (data are shown as means ± SEM, n = 3). **** demonstrates significant differences between biological conditions by *p* < 0.0001.

Supporting the above observations, ZIKV load was also shown to be high in HASRT/ci35-infected cells in comparison with CCF-STTG1 cells for both MR766 and PRVABC59 (Figure 2A,B). Similarly, ZIKV RNA load was also significantly higher in MR766-strain-infected hTERT cells compared with the PRVABC59 strain (Figure 2C). Examination of viral load in the culture media at 24 h post-infection with MR766 demonstrated that the increased viral replication observed in the HASTR/ci35 cell line compared with the CFF-STGG1 cell line at this timepoint (Figure 2A,B) corresponded to an increase in extracellular viral particle release (Figure 2D). However, the foetal-hTERT astrocytes were demonstrated to also have reduced viral particles in the cellular supernatant at 24 h post-infection despite the observed increase in viral proteins intracellularly in Figure 1C, perhaps indicating that ZIKV is spreading more successfully intercellularly; further experiments would be required to validate this. Lastly, in order to compare whether or not viral entry restrictions could account for the reduced viral load in some astrocyte cell lines, viral RNA was compared between the CFF-STGG1 and the HASTR/i35 cells at 6 h post-infection to give a measure of viral entry. As can be seen in Figure 2E, viral entry of both MR766 and PRVABC59 was relatively equivalent in both CCF-STTG1 and HASTR/ci35 cells, demonstrating that other cellular mechanisms were involved in cellular control of ZIKV replication.

### 3.2. Comparable Expression Level of Known Antiviral Genes in Resistant and Susceptible Astrocyte Cell Models

Virus infection of the host cell induces antiviral response via the activation of different mechanisms where pattern recognition receptors detect viral nucleic acid and other material (PAMPS, pathogen-associated molecular patterns) to initiate a signalling cascade which culminates in the production of interferons, the main antiviral cytokines [38,39]. These interferons then subsequently activate the JAK–STAT pathway to upregulate hundreds of interferon-stimulated genes (ISGs), many of which are considered to be directly antiviral [40,41]. To understand the antiviral gene expression features of the most ZIKV-resistant and ZIKV-susceptible astrocyte cell models, we next compared gene expression patterns of the CCF-STTG1 and HASTR/ci35 cells following ZIKV infection at different timepoints.

**Figure 3:**
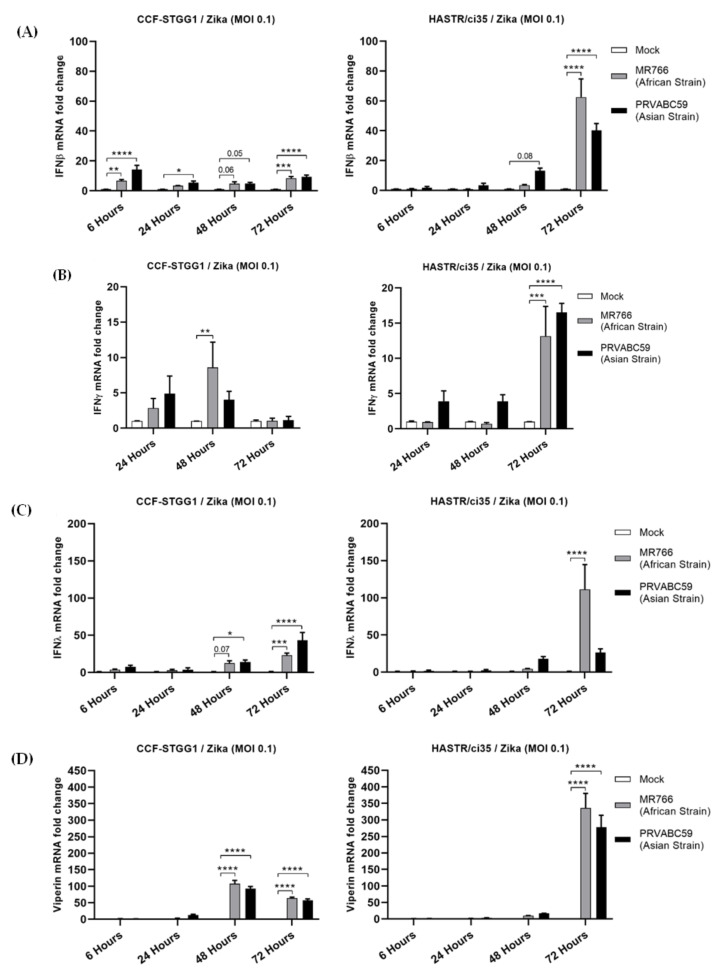
Comparable expression of type I (IFN-β) (**A**), II (IFN-γ) (**B**), III (IFN-λ) (**C**) interferons and a broad-spectrum antiviral inter-feron-stimulated gene (ISG) viperin (**D**) in resistant (CCF-STTG1) and susceptible (HASTR/ci35) astrocyte cells following ZIKV infection. Both cells were infected at an MOI of 0.1 and mRNA expression was measured at 6-, 24-, 48- and 72-hour timepoints. Data were normalised to the 36b housekeeping gene and expressed as a fold change relative to mock-infected control (data are shown as means ± SEM, *n* = 3). *, **, *** and **** demonstrate significant differences between biological conditions by *p* < 0.05, *p* < 0.01, *p* < 0.001 and *p* < 0.0001, respectively.

Both cell lines examined displayed minimal expression of type I IFN in the early timepoints following ZIKV infection with either strain (Figure 3A). However, the more ZIKV-resistant CFF-STGG1 cell model demonstrated comparatively higher expression of type I IFN (Figure 3A, fold change 6.76 (MR766) and 14.23 (PRVABC59)) compared with the ZIKV-susceptible HASTR/ci35 cells (Figure 3A, fold change 1.06 (MR766) and 1.98 (PRVABC59)) at the early (6-hour) timepoint despite the lower levels of virus infection (Figure 1A and Figure 2A,B), with the HASTR/ci35 astrocyte cell line only demonstrating a significant increase in type I IFN at the later timepoints. Similarly, IFN-β cytokine production was comparable among all three astrocytes at 48 h of MR766 strain post-infection, but IFN-β production was higher in HASTR/ci35 and hTERT astrocytes compared with CCF-STTG1 cells at 72 h post-infection (Figure S1) consistent with the trend in IFN-β mRNA expression dynamics (Figure 3A). The induction of type III IFN (IFN-λ) mRNA following ZIKV infection followed an identical trend to type I IFN (Figure 3C), perhaps indicating that astrocytes do not preferentially induce either of the IFNs following ZIKV infection.

Type II interferon, represented only by IFN-γ, can also inhibit the growth of viral and other pathogenic infections [42,43]. Our data demonstrate limited upregulation of IFN-γ expression in both cell types following ZIKV infection in comparison with the levels of both type I and III IFNs; however, the more ZIKV-susceptible HASTR/ci35 astrocyte cells did demonstrate a peak of mRNA expression for type II IFN at the later timepoints (Figure 3C), coinciding with maximal virus infection.

All three types of IFNs induce the expression of IFN-stimulated genes (ISGs) which are crucial for the inhibition of viral infection, replication, or egress; some may act on more than one stage of the virus replication cycle. To assess the ability of the production of IFN mRNA to drive the expression of interferon-stimulated genes (ISGs), we also examined the upregulation of viperin mRNA (Figure 3D). Among the antiviral ISGs, viperin, also known as RSAD2 (radical SAM domain-containing 2) is a well characterised antiviral gene and was demonstrated as one of the major host restriction factors in controlling different virus infections [44,45,46,47], including ZIKV [48]. Interestingly, viperin mRNA was significantly induced much earlier in the ZIKV-resistant CFF-STGG1 cells (fold change 107.71 (MR766) and 92.93 (PRVABC59)) at 48-hour in response to ZIKV infection (Figure 3D), once again, despite the lowered viral replication in this cell line (Figure 1A and Figure 2A,B), but reached higher expression levels in the ZIKV-susceptible astrocytes (HASTR/ci35) at the later timepoint, demonstrating a delayed response in comparison with the more ZIKV-resistant astrocyte cells (Figure 3D, fold change 336.02 (MR766) and 277.89 (PRVABC59)).

### 3.3. Inflammatory Cytokines Expression in Resistant and Susceptible Astrocyte Cell Model following ZIKV Infection

**Figure 4:**
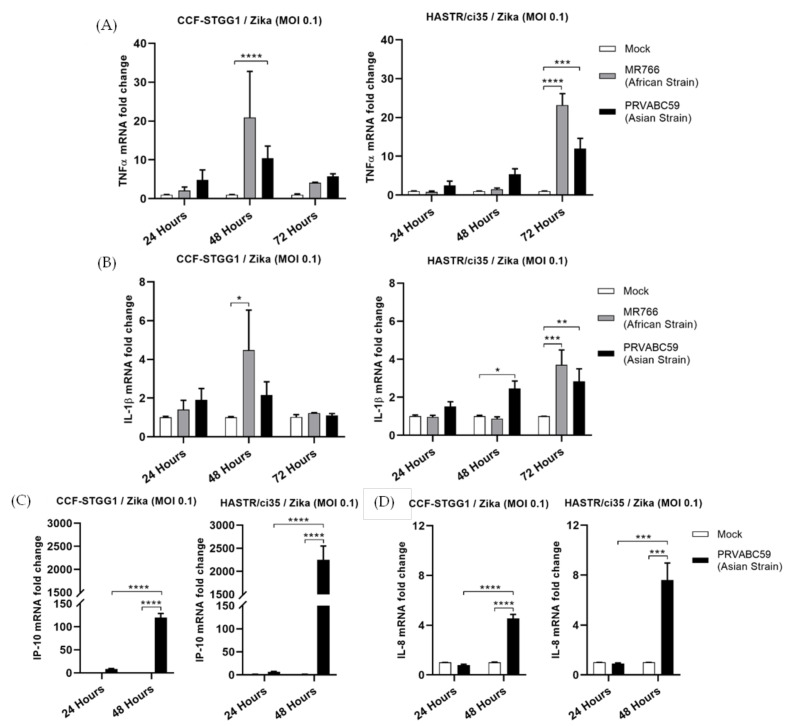
Proinflammatory (TNFα and IL-1β) and inflammatory (IP-10 and IL-8) genes showed a comparable expression pattern especially at earlier timepoints of ZIKV post-infection in CCF-STTG1 and HASTR/ci35 astrocyte cells. Cells were infected at an MOI of 0.1 and mRNA expressions were assessed at 24- and 48-h timepoints. Data were normalised to the 36b housekeeping gene and expressed as a fold change relative to mock-infected control (data are shown as means ± SEM, n = 3). Statistical analyses between two (**C**,**D**) and multiple (**A**,**B**) biological conditions were performed using one- and two-way ANOVA comparisons with Tukey and Sidak’s post hoc tests, respectively. *, **, *** and **** demonstrate significant differences between biological conditions by *p* < 0.05, *p* < 0.01, *p* < 0.001 and *p* < 0.0001, subsequently.

During activation of the early innate antiviral signalling pathways, following viral PAMP recognition, the transcription factor NF-kB can become activated and is responsible for driving an inflammatory gene signature [49,50]. To determine potential differences in the mRNA expression of pro-inflammatory cytokines, TNFα and IL-1β along with the chemokine IP-10 and inflammatory cytokine IL-8 were assessed in our cell lines following ZIKV infection. RT-qPCR analysis of the abundance of transcripts for TNF-α or IL-1β showed very low levels of activation of these pro-inflammatory cytokines in either cell type following ZIKV infection (Figure 4A,B), with the levels once again appearing to follow the time-course study of viral infection in each cell line (Figure 2D,E), as was seen for IFN-γ mRNA expression (Figure 3B). Comparable levels of IL-8 were also observed in both astrocyte cell lines (Figure 4D); however, expression levels of the chemokine, IP-10 were significantly elevated in both cell types following ZIKV infection, with much higher levels seen in the more ZIKV-susceptible HASTR/ci35 cells at 48-hour p.i. IP-10 is also inducible following the expression of type I IFN [51], and it is potentially this pathway that is driving its expression in this instance, given the relatively low mRNA levels of TNF-α and IFN-γ following PRVABC59 infection at a similar timepoint; both alternate drivers of IP-10 expression [52,53,54].

### 3.4. Pre-Stimulation of ZIKV-Susceptible Astrocytes with Viral Mimics or Interferon Can Alter the Outcome of ZIKV Infection

To assess the ability of either the main antiviral cytokines, IFN-β (type I) and IFN-λ (type III), or viral mimic activators of early antiviral innate immunity towards viral infection in our ZIKV-susceptible astrocyte cells, HASTR/ci35 cells were pre-treated with IFNs or dsRNA/dsDNA analogues for 18 h prior to infection with ZIKV (PRVABC59 strain).

**Figure 5:**
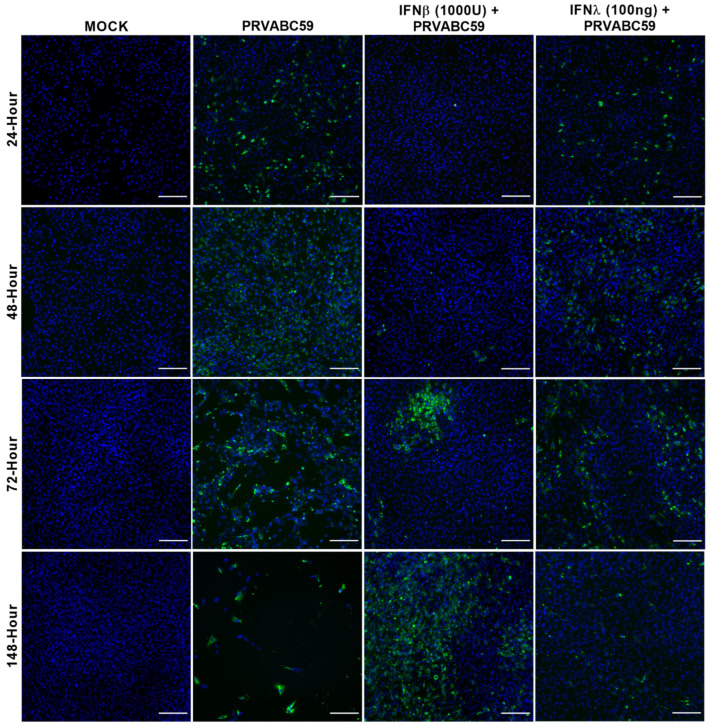
IFN-β and IFN-λ pre-stimulated ZIKV-susceptible HASTR/ci35 astrocyte cells partially control Zika virus strain PRVABC59 infection. Subconfluent ZIKV-susceptible HASTR/ci35 astrocyte cells were incubated with a low concentration of IFN-β (1000 U/mL) and IFN-λ (100 ng/mL) for 18 h followed by inoculation with PRVABC59 ZIKV strain at an MOI of 0.1. IFN-β showed effective control of the virus at an earlier timepoint but was unable to control persistently, whereas IFN-λ showed persistent but partial control of the virus. Cells were fixed at 24-, 48-, 72- and 148-h timepoints and then stained with 4G2 antibody to detect ZIKV envelope protein Env (stained green) and DAPI stain for cell nuclei (stained blue). Scale bars represent 200 µm.

Dosages of IFN-β and IFN-λ were selected based on previous studies, with IFN-β pre-treatment of 1000 and 2000 U/mL found to be the most effective in many treatment studies [55,56,57,58], and an IFN-λ pre-dose ranging from 100 ng/mL to 300 ng/mL being previously used in several studies [59,60]. As can be seen in Figure 5, pre-treatment with neither type I (IFN-β) nor type III (IFN-λ) IFN was found to be fully protective against viral infection in the ZIKV-susceptible HASTR/ci35 astrocytes (Figure 5 and Figure S2). Type I IFN was shown to delay the ZIKV infection of the cells and lengthen their survival, and type III IFN (IFN-λ) was seen to control viral infection to some extent but only at the later time of infection (at 148-h post-infection) (Figure 5). However, this limited ZIKV control at the later time of infection in the type III IFN pre-treated cells resulted in the cells being less susceptible to cell death. Furthermore, pre-treatment with higher IFN-β concentration (2000 U/mL) showed better control of ZIKV infection than a lower concentration (Figure 5 and Figure S2), but unlike IFN-β pre-treatment, no dose-dependent variation in ZIKV control was observed between the high and low amounts of IFN-λ pre-treated cells (Figure 5 and Figure S2). These results indicate that the production of type I or III IFN by the astrocyte cells following ZIKV infection is not likely to be the limiting factor alone in cellular infection levels.

**Figure 6:**
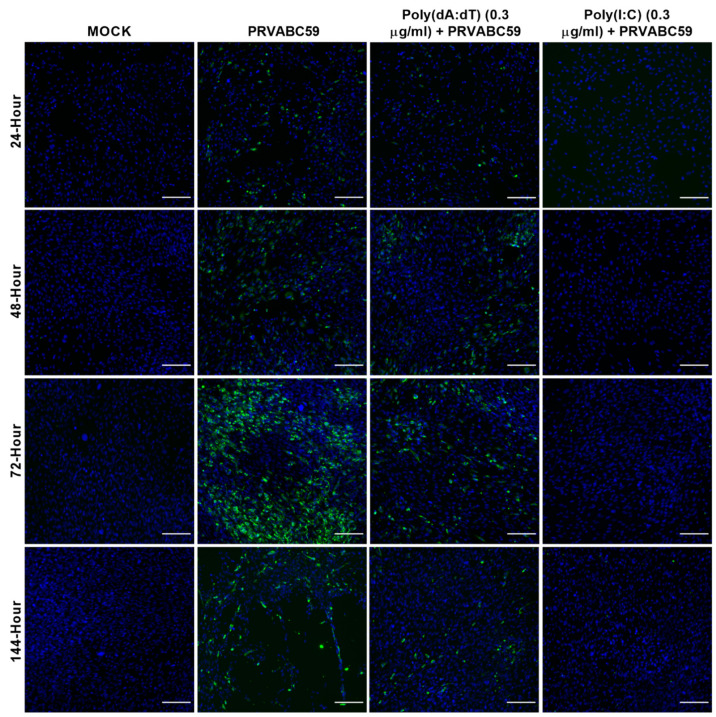
The 0.3 µg/mL poly(dA:dT) and poly(I:C) pre-stimulated HASTR/ci35 astrocyte cells are able to control Zika virus strain PRVABC59 infection. Subconfluent ZIKV-susceptible HASTR/ci35 astrocyte cells were transfected with low concentrations (0.3 µg/mL) of poly(I:C) and poly(dA:dT) 18 h prior to infection with PRVABC59 ZIKV strain at an MOI of 0.1. Poly(I:C) showed complete control of the virus at this very low concentration whereas poly(dA:dT) was relatively less effective at this concentration. Cells were fixed at 24-, 48-, 72- and 144-h timepoints and then stained with 4G2 antibody to detect ZIKV envelope protein Env (stained green) and DAPI stain for cell nuclei (stained blue). Scale bars represent 200 µm.

**Figure 7:**
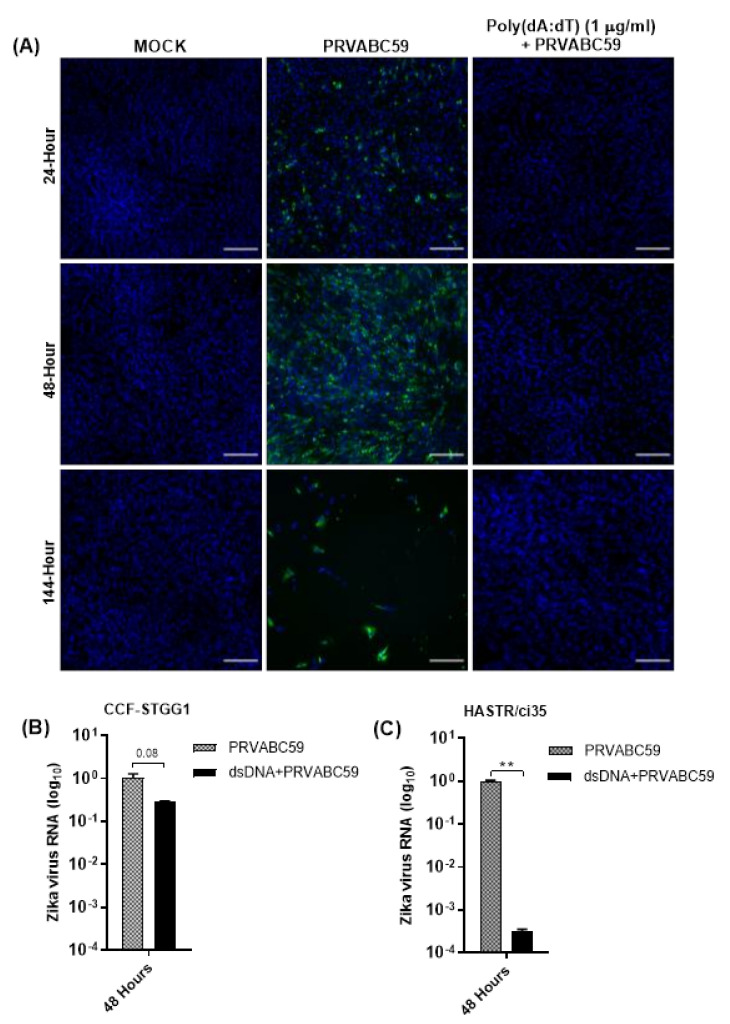
(**A**) Pre-stimulation of HASTR/ci35 astrocyte cells with higher concentration (1 µg/mL) of poly(dA:dT) completely controlled PRVABC59 infection, unlike lower (0.3 µg/mL) concentration of poly(dA:dT) pre-stimulation which did not show complete control (Figure 6). (**B**,**C**) Zika virus RNA load in poly(dA:dT) pre-stimulated CCF-STTG1 and HASTR/ci35 cells. Subconfluent ZIKV-susceptible HASTR/ci35 astrocyte cells were transfected with 1 µg/mL of poly(I:C) or poly(dA:dT) 18 h prior to infection with PRVABC59 ZIKV strain at an MOI of 0.1. (**A**) Cells were fixed at 24-, 48- and 144-h timepoints and then stained with 4G2 antibody to detect ZIKV envelope protein Env (stained green) and DAPI stain for cell nuclei (stained blue). Scale bars represent 200 µm. (**B**,**C**) Cell lysates were collected for relative quantification of viral RNA load at 48-h timepoint. Data were normalised to the 36b housekeeping gene and viral fold change at 48-h timepoint is expressed as a fold change relative to ZIKV RNA load at 6 hr timepoints. Viral RNA reduction (fold change) in poly(dA:dT) pre-stimulated cells was calculated considering the average viral RNA fold change compared with Zika-infected cells at 48-h timepoint. Unpaired T-test was performed to identify significant differences between two experimental conditions. ** demonstrates significant differences between biological conditions by *p* < 0.01.

The activation of both TLR3 and RIG-I pathways has been demonstrated to be protective in the infection of host cells with ZIKV, with the former being shown previously to be protective in an alternative astrocyte model of ZIKV infection [61,62]. Additionally, it is known that ZIKV inactivates the STING pathway via direct cleavage of STING (Ding et al. 2018); however, a role for STING-activated gene sets in the protection of host cells against ZIKV has not previously been shown in humans. Therefore, we sought to dissect the pre-treatment of our ZIKV-susceptible astrocyte cell model with poly(dA:dT), and compare and contrast their responses to viral infection (Figure 6 and Figure 7). Pre-treatment of cells with 0.3 μg/mL poly(I:C) was able to completely protect ZIKV-susceptible astrocytes against viral infection; however, poly(dA:dT) was only slightly protective at this concentration (Figure 6).

To investigate the dose-dependency of poly(dA:dT)-mediated ZIKV control, susceptible astrocyte cells were also pre-stimulated with 1 μg/mL poly(dA:dT), 18 h prior to infection (Figure 7A). Interestingly, when the pre-treatment amounts of poly(dA:dT) were increased to 1 μg/mL, complete protection was observed (Figure 7A). To further validate the protection of astrocytes from ZIKV infection following poly(dA:dT) pre-treatment, the presence of viral RNA in both ZIKV-susceptible HASTR/ci35 and ZIKV-resistant CCF-STTG1 astrocyte cells was assessed (Figure 7B,C). Poly(dA:dT) demonstrated high efficiency in controlling ZIKV infection in both cell types, with viral RNA loads in poly(dA:dT) pre-treated ZIKV-susceptible HASTR/ci35 astrocytes being comparable with poly(dA:dT) pre-treated ZIKV-resistant CCF-STTG1 astrocytes. However, this may not be representative in Figure 7B,C, because Zika virus load in CCF-STTG1 astrocyte cells is already low to start with and a value of 1 was assigned for average viral RNA fold change at the 48-hour timepoint for both CCF-STTG1 and HASTR/ci35 astrocytes, regardless of viral load at that time. Importantly, Figure 7B,C demonstrates the efficient reduction of ZIKV RNA load in astrocytes cells when prestimulated with poly(dA:dT) mimic regardless of the presence of low and high amounts of virus in CCF-STTG1 and HASTR/ci35 astrocytes, respectively. These findings indicate that the inability of the ZIKV-susceptible HASTR/ci35 cells to control ZIKV infection is not because of an intrinsic inability to mount an antiviral response, but rather the inability to mount a proper anti-ZIKV response at the earlier stage of infection.

### 3.5. Antiviral and Inflammatory Gene Expression following poly(dA:dT) Pre-Stimulation in Astrocyte Cell Models

**Figure 8:**
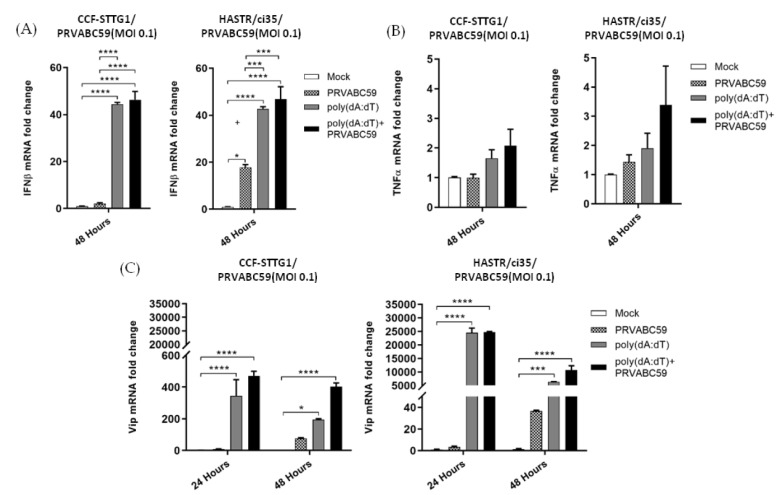
Expression of antiviral and inflammatory genes in poly(dA:dT) pre-stimulated, and ZIKV-infected CCF-STTG1 and HASTR/ci35 cells. Antiviral and proinflammatory gene expression following stimulation with poly(dA:dT) following PRVABC59 or mock infection. Subconfluent ZIKV-resistant CCF-STTG1 and -susceptible HASTR/ci35 cells were transfected with 1µg/mL poly (dA:dT) 18 h prior to infection with PRVABC59 strain. Cell lysates were collected at either 48-hour (**A**,**B**) or 24- and 48-h timepoints (**C**) to assess relative mRNA expression of major antiviral (IFN-β and viperin) and inflammatory (TNFα) genes. Both astrocyte cell types showed similar patterns in expression of the above-mentioned genes. Data were normalised to the 36b housekeeping gene and expressed as a fold change relative to mock-infected control (data are shown as means ± SEM, *n* = 3). *, *** and **** demonstrate significant differences between biological conditions at the level of *p* < 0.05, *p* < 0.001 and *p* < 0.0001, respectively.

As the viral mimic pre-treatment of both astrocyte cell models resulted in reduced ZIKV load, we chose to investigate the downstream expression of antiviral (IFN-β) and pro-inflammatory (TNF-α) cytokines, along with the known anti-ZIKV host protein, viperin, in pre-treated astrocytes in the presence or absence of ZIKV infection. For these experiments, astrocyte cells from both cell models were pre-treated with 1 µg/mL of both poly(dA:dT) or poly I:C 18 h prior to ZIKV infection. However, pre-treatment with 1 µg/mL poly(I:C) displayed considerable cell death, perhaps being toxic for these cell models at this concentration, so the data are not presented. Poly(dA:dT) pre-treatment resulted in a similar level of upregulation of IFN-β in both cell types, with a much enhanced upregulation of IFN-β seen following pre-treatment of the cells; however, ZIKV infection did not significantly upregulate IFN-β mRNA levels further (Figure 8A). Not surprisingly, viperin mRNA was observed to act in a similar fashion to that seen for IFN-β mRNA, with pre-treatment of the cells with poly(dA:dT) significantly enhancing viperin mRNA to a much greater extent than viral infection alone, with no further upregulation seen upon viral infection following pre-stimulation (Figure 8C). Notably, in both cell models, minimal mRNA increases were seen for TNF-α, despite the treatment grouping (Figure 8B). Collectively, these results indicate that pre-stimulation with poly(dA:dT) offers a protective antiviral effect upon ZIKV infection of susceptible astrocyte cells for the first time, but does not offer a conclusive mechanism for this protection.

## 4. Discussion

Zika virus (ZIKV) is reported to infect the brain of both foetuses and adults and is known to cause detrimental ZIKV-associated neurological complications, such as microcephaly in infants [3,63,64], and encephalitis, myelitis, and meningoencephalitis in adults [65,66,67,68]. Following the recent outbreak in 2015 in Brazil, in 2016 in 31 countries in the Americas, and with increasing evidence of the link between Zika virus (ZIKV) infection of the central nervous system (CNS) and microcephaly in newborns, a significant number of studies have been performed to understand Zika virus epidemiology, evolution, viral biology, structure, replication, transmission, infection models, and pathogenesis, and host immune response to Zika virus infection. The host response to Zika virus infection in the CNS has been one of the most focused areas of Zika virus research, since it causes multiple neurological disorders in the CNS. However, the full spectrum of host immune responses in the CNS following Zika infection is yet to be explored, as ZIKV can infect a range of host cells in the CNS, leading to the induction of a variety of host responses [22,23,25]. Multiple recent studies have investigated the astrocyte response to ZIKV infection, given it is the most abundant cell type of the CNS and was shown to be resistant to infection by multiple neurotropic flaviviruses, including Zika virus, even though some astrocytes can harbour low-level infection by these viruses [16,17,18]. Interestingly, ZIKV has also been demonstrated to infect human foetal astrocytes persistently by delaying apoptosis, regardless of the presence of a highly active antiviral response [23]. To explain the reported variable ability of the astrocytes to control ZIKV, a more detailed understanding of astrocyte–ZIKV interactions is necessary, which may facilitate the development of novel and effective anti-ZIKV therapeutics that can be used in patients with different physiological statuses. Therefore, in this study we further elucidated the interactions between various human astrocyte cell models and ZIKV, by assessing the astrocyte response to ZIKV infection in some newly developed astrocyte models, two of which have foetal origins; CCF-STTG1, hTERT human primary immortalised and HASTR/ci35 astrocytes.

Our studies demonstrated divergence between astrocyte responses to ZIKV infection across the three astrocyte cell models, with the CCF-STTG1 astrocyte cell model displaying a high level of ZIKV resistance, in comparison with the HASTR/ci35 astrocyte cell model, which underwent almost complete cell death by 72 h post-infection (Figure 1A,B and Figure 2A,B); these observations were similar despite the ZIKV strain. Interestingly, the foetal-hTERT primary immortalised astrocytes also displayed high resistance to the PRVABC59 ZIKV strain, but not to the MR766 ZIKV strain, which synchronises with previous reports of MR766 being able to infect mammalian cells at a higher rate [69,70].

To examine the reason for the varying ability of these astrocyte models to control Zika virus infection, gene expression studies were performed following ZIKV infection. Although both astrocyte cell lines expressed IFN-β mRNA, the expression levels were significantly higher in the more ZIKV-resistant CCF-STTG1 cells very early post-infection, despite this astrocyte cell line demonstrating slightly lower levels of ZIKV RNA. The more ZIKV-susceptible HASTR/ci35 cells only demonstrated significant upregulation of IFN-β mRNA at 48 h post-infection. A similar scenario was also observed in IFN-λ mRNA expression, as well as in the expression of the antiviral restriction factor, viperin, perhaps indicating that IFN production alone is not the varying factor between these cell subtypes in respect to differential control of ZIKV infection; however, the pace of type I and III IFNs expression may be a factor. This observation is in line with a previous report suggesting that a rapid type I IFN response may protect murine astrocytes from cell death following flavivirus infections including ZIKV [17]; however, future work in understanding the ability of one astrocyte cell line to control ZIKV infection over a similar cell line may add important knowledge in regard to further cellular host factors in control of this rapid IFN response.

To improve the understanding of the potential role of the inflammatory response to control Zika virus infection in astrocytes, the expression of proinflammatory and inflammatory genes was assessed along with type II interferon (IFN-γ) expression. IFN-γ was expressed at a low level in both cell types; however, expression was controlled by the more ZIKV-resistant CCF-STTG1 cells at the later timepoint (Figure 3B). Similar patterns of mRNA expression were also observed for TNF-α, IL-1β, IL-8 and CXCL-10 (IP-10) cell models following ZIKV infection; however, with a much higher level of IP-10 expression observed in the ZIKV-susceptible HASTR/ci35 cells compared with the ZIKV-resistant CCF-STTG1 cells. Overall, the ZIKV-resistant CCF-STTG1 cells appear to better control the cellular inflammatory response in comparison with the more ZIKV-susceptible HASTR/ci35 cells at later timepoints of infection, which synchronises with ZIKV-mediated cell death in the susceptible cell line at the later time of infection.

There are diverse cellular antiviral mechanisms that act against specific viral infections, including both interferon-dependent and interferon-independent mechanisms. Interferon-dependent antiviral pathways can be divided based on the type of interferon and interferon-stimulated genes acting against a specific virus infection. To examine the ability of IFNs to control ZIKV infection in astrocyte cells, IFN type I (IFN-β) and type III (IFN-λ) were assessed for their anti-ZIKV ability, because they have the ability to drive different sub-sets of interferon-stimulated genes, many of which are antiviral [71,72,73]. Our data demonstrate reduced ZIKV infection in the IFN-β pre-stimulated susceptible astrocyte (HASTR/ci35) cells at earlier times of post-infection, but the control of Zika replication declined over time (Figure 5 and Figure S2). The control of ZIKV infection in IFN-β pre-treated susceptible astrocyte cells varied depending on the IFN-β concentration used, suggesting that higher doses of IFN-β pre-stimulation might better control the ZIKV infection. Type III IFN has been reported to be produced by astrocytes following ZIKV infection and is known to control HSV-1 infection in astrocytes, but there is limited knowledge on its role in the control of ZIKV infection [74,75]. Our data demonstrate persistent ZIKV infection in susceptible HASTR/ci35 astrocyte cells pre-treated with IFN-λ; however, cell death was significantly reduced compared with untreated cells (Figure 5), and this pattern remained unchanged with higher concentrations of IFN-λ (Figure S2). These findings are suggestive that astrocyte control of Zika virus infection does not depend solely on the expression of IFN-β or IFN-λ.

As the expression of ISGs can also occur independently of IFN expression [76,77], we sought to simulate a potentially more diverse range of antiviral proteins using viral mimics. Interestingly, poly(I:C) pre-stimulation demonstrated complete control of ZIKV infection in ZIKV-susceptible astrocyte cells, whereas poly(dA:dT) pre-stimulation of astrocytes showed partial control of ZIKV infection at lower concentrations (Figure 6), but complete control at a higher concentration (Figure 7). Our data from our poly(I:C) pre-stimulation studies support previous studies that demonstrated the protection of host cells from ZIKV infection following the activation of TLR3, RIG-I and TLR7/8 pathways [61,62,78]. A previous study demonstrated the species-specific disruption of STING-dependent antiviral cellular defences via the direct cleavage of STING by the ZIKV NS2B3 protease [79]; however, the role for STING-activated gene sets in the protection of host cells against ZIKV has not been demonstrated previously. Pre-stimulation of astrocytes with poly(dA:dT) resulted in similar levels of upregulation of IFN-β mRNA in both astrocyte cell types, with a greater upregulation of IFN-β seen in poly(dA:dT) pre-treated cells compared with ZIKV infection alone; however, pre-stimulation followed by ZIKV infection was not able to further upregulate IFN-β mRNA. A similar pattern of mRNA expression was observed for the viperin gene, and again with much higher mRNA expression levels seen in poly(dA:dT) pre-treated cells than in viral infection alone, and no further upregulation was seen upon viral infection following pre-stimulation with poly(dA:dT). Notably, negligible amounts of TNF-α mRNA upregulation were observed in both astrocyte cell models following the poly(dA:dT) pre-stimulation, perhaps indicating that targeting a dsDNA activation pathway in astrocytes would be useful in a situation where a higher antiviral response but controlled inflammatory response is necessary to control a viral infection; however, further work would be required to fully understand this process.

Overall, this study contributes to the current knowledge of astrocyte-mediated Zika infection control, by characterising the responses to ZIKV infection in susceptible and resistant astrocyte cell models. More specifically, findings in this study improve our understanding of the variability in astrocyte response following ZIKV infection and suggest an involvement of interferon type I- and III-independent antiviral pathways in Zika infection control of astrocytes. Additionally, for the first time, we demonstrate that type III IFN appears to be partially protective against ZIKV infection in astrocytes which remained unchanged throughout the timecourse, further highlighting the potential variation in the antiviral gene sets that these two cytokines can induce despite their common use of the JAK–STAT pathway. This study also demonstrates, for the first time, the ability of poly(dA:dT) pre-stimulation or activation of the dsDNA recognition pathway to mount a successful anti-ZIKV response in susceptible astrocytes, even though the Zika virus is an RNA virus, and reconfirms astrocyte’s ability to control ZIKV infection when pre-stimulated with poly(I:C), a viral dsRNA mimic. Furthermore, the identification of Zika virus-resistant CCF-STTG1 and ZIKV-susceptible HASTR/ci35 astrocyte models will further allow researchers to study astrocyte-mediated responses to other viral infections that occur in the central nervous system.

## Data Availability

The data presented in this study are available on request from the corresponding author.

## Supplementary Materials

The following are available online at https://www.mdpi.com/article/10.3390/biology11010143/s1, Figure S1: Comparable production of type -I (IFN-β) interferon in resistant (CCF-STTG1), and susceptible (HASTR/ci35) and hTERT (Abmgood) astrocyte cells following ZIKV strain MR766 infection at 48 h post infection title, Figure S2: Pre-stimulation of ZIKV susceptible HASTR/ci35 astrocyte cells with higher concentration of IFN-β and IFN-λ also showed partial control of the PRVABC59 strain similar toprestimulation with lower concentration shown in Figure 4, Table S1: List of real time qPCR primer sequences used in this study.
