# Supplementary material for: Astrocyte Control of Zika Infection Is Independent of Interferon Type I and Type III Expression
Source: Biology (Basel). 2022 Jan 15;11(1):143. doi: 10.3390/biology11010143 (PMC8772967; doi:10.3390/biology11010143)
Supplement: Supplementary file 1 [file biology-11-00143-s001.zip › biology-1497307-supplementary.pdf]

biology MDPI

Supplementary Materials:

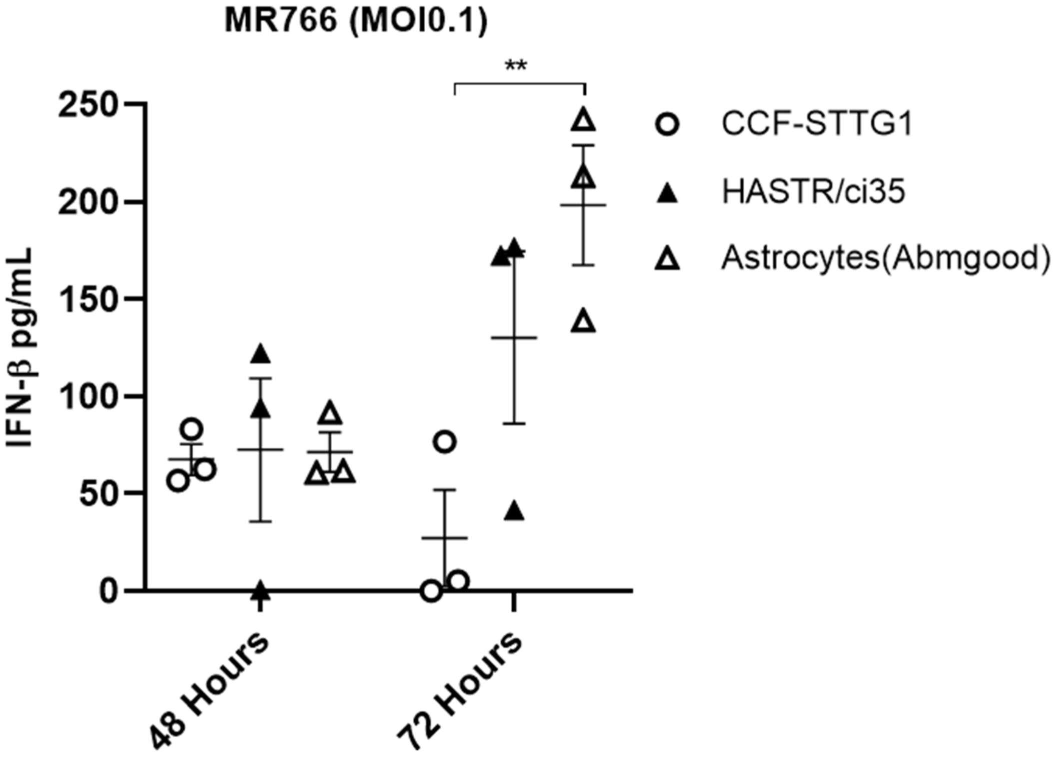

Figure S1. Comparable production of type -I (IFN-β) interferon in resistant (CCF-STTG1), and susceptible (HASTR/ci35) and hTERT (Abmgood) astrocyte cells following ZIKV strain MR766 infection at 48 hours post infection. All cells were infected at an MOI of 0.1 and IFN-β cytokine production was measured at 48-and 72-hour timepoints using ELISA assay. Data are shown as means ± SEM, n = 3. ** demonstrates significant differences between two biological conditions by $p < 0.01$.

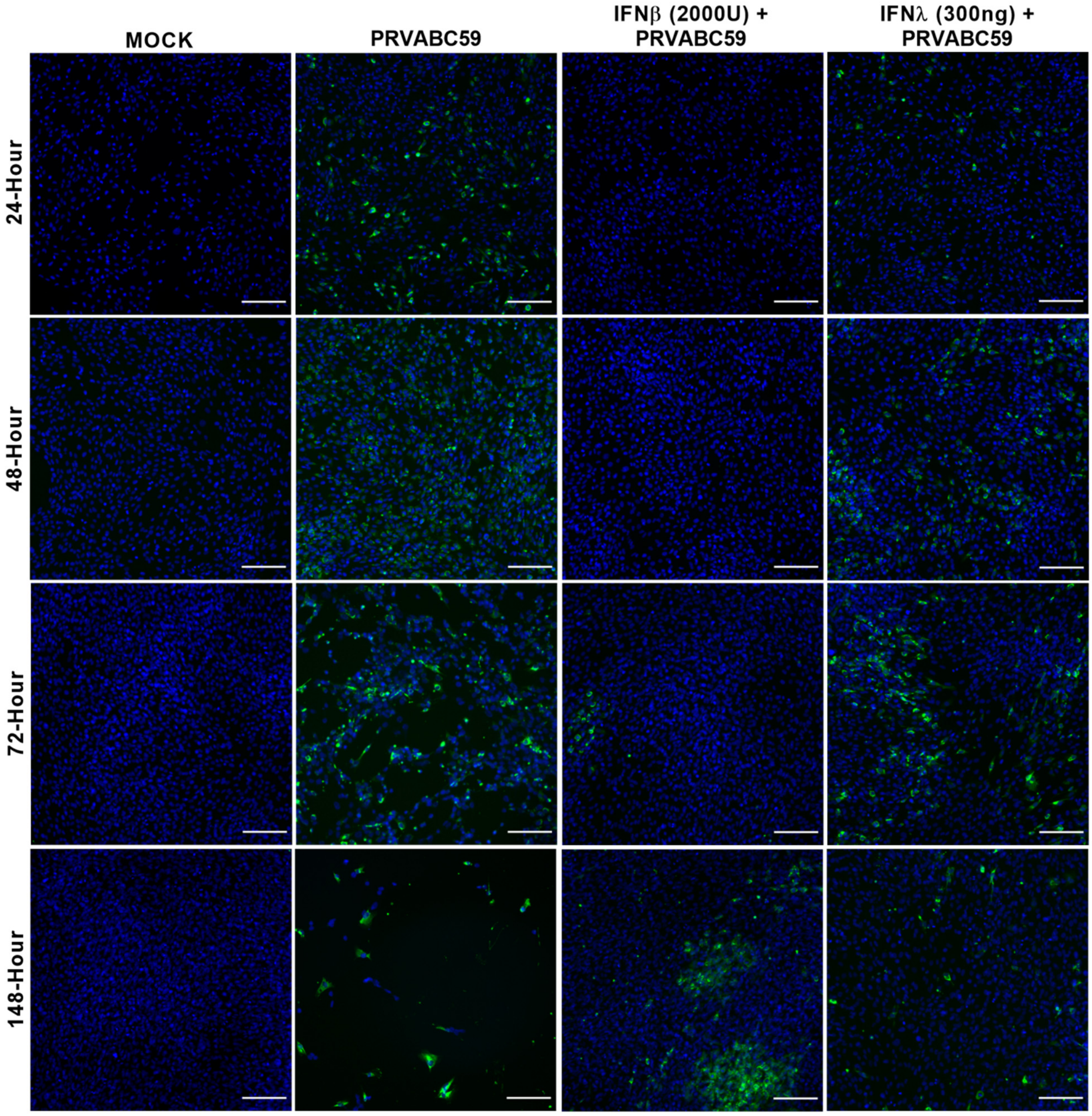

Figure S2. Pre-stimulation of ZIKV susceptible HASTR/ci35 astrocyte cells with higher concentration of IFN-β and IFN-λ also showed partial control of the PRVABC59 strain similar toprestimulation with lower concentration shown in Figure 4. Subconfluent ZIKV susceptible HASTR/ci35 astrocyte cells were incubated with comparatively higher concentration of IFN-β (2000U/ml) and IFN-λ (300ng/ml) for 18 hours followed by inoculation with PRVABC59 ZIKV strain at an MOI of 0.1. Similar to the data shown in Figure 4 IFN-β showed effective control of the virus at earlier timepoints but was unable to control persistently whereas IFN-λ showed persistent but partial control of the virus. Cells were fixed at 24-, 48-, 72- and 148-hour timepoints and then stained with 4G2 antibody to detect ZIKV envelope protein Env (stained green) and DAPI stain for cell nuclei (stained blue). Scale bars represent 200 μm.

Table S1. List of real time qPCR primer sequences used in this study.

Gene/Transcript Name	Forward Primer (5' to 3')	Reverse Primer (5' to 3')
Zika virus	CAGCTGGCATCATGAAGAAYC	CACYTGTCCCATCTTYTTCTCC
IFN-β	TGTCAACATGACCAACAAGTGTCT	GCAAGTTGTAGCTCATGGAAAGAG
IFN-λ	GGAAGAGTCACTCAAGCTGAAAAA	AGAAGCCTCAGGTCCCAATCC
IFN-γ	TCCTGTGACTGTCTCACTTAATC	CTTAGGTTGGCTGCCTAGTT
Viperin	AATTGAATTCATGTGGGTGCTTACACCTGCTG	AATAGGATCCCTACCAATCCAGCTTCAGATCA
TNF-α	CAGGTTCTCTTCCTCTCACATAC	GTCCCGGATCATGCTTTCA
IL-1β	ATGGACAAGCTGAGGAAGATG	CCCATGTGTCGAAGAAGATAGG
CXCL10	TCCACGTGTTGAGATCATTGC	TCTTGATGGCCTTCGATTCTG
CXCL-8	TGTGCCTTGGTTTCTCCTTTA	AAGTGCTTCCACATGTCCTC
